# Supplementary figures and images for: Use of next generation sequencing to compare simple habitat and species level differences in the gut microbiota of an invasive and native freshwater fish species
Source: PeerJ. 2020 Dec 18;8:e10237. doi: 10.7717/peerj.10237 (PMC7751434; doi:10.7717/peerj.10237)

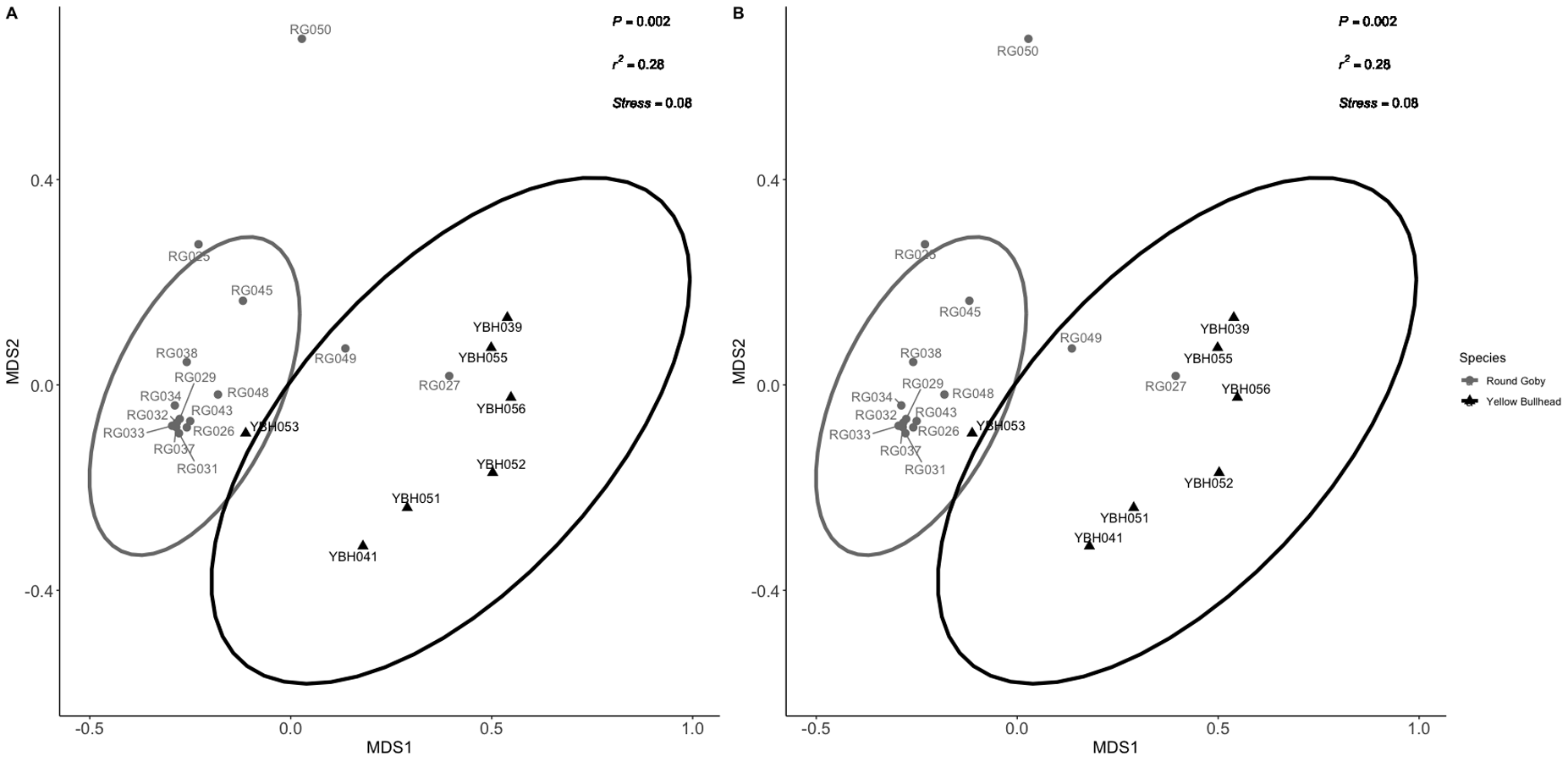

Supplement: Supplemental Information 1 — Procrustes analysis revealed significant similarity between the two ordinations (P = 0.001). [file peerj-08-10237-s001.png]

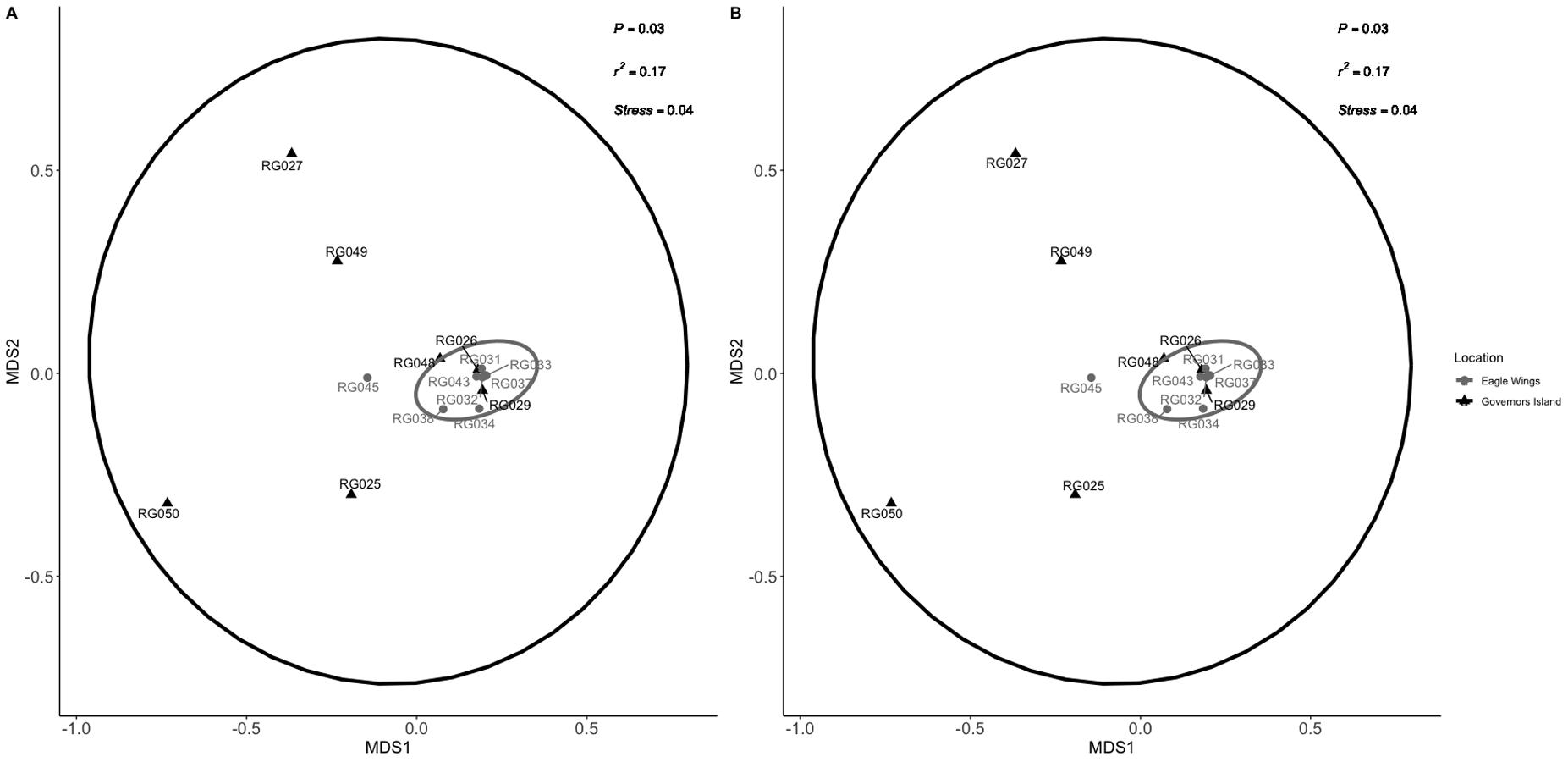

Supplement: Supplemental Information 2 — Procrustes analysis revealed significant similarity between the two ordinations (P = 0.001). [file peerj-08-10237-s002.png]

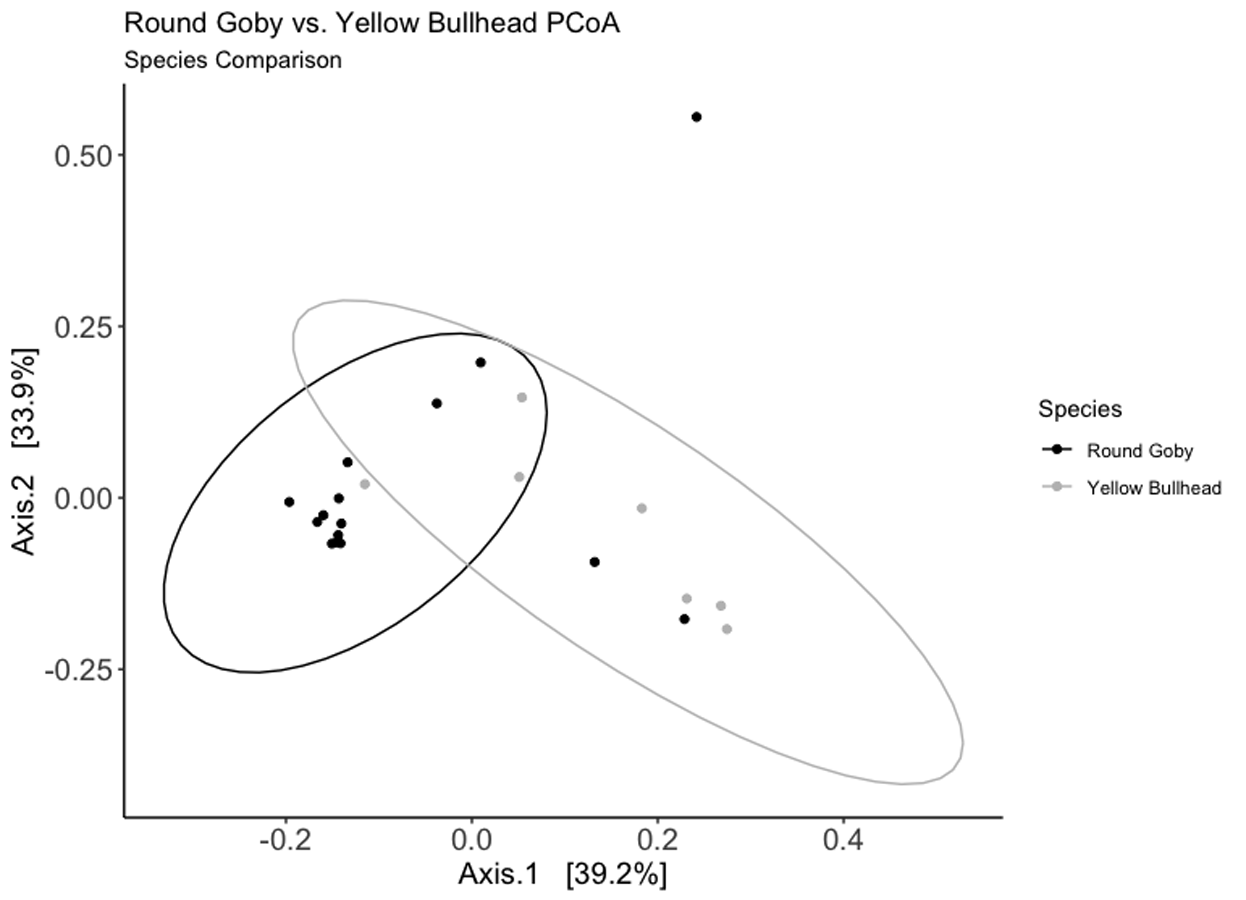

Supplement: Supplemental Information 3 — Axes 1 +2 combine for over 73% of the total data variation. The shape of the PCoA ordination closely mimics that of the species NMDS ordination calculated using Bray–Curtis distances. [file peerj-08-10237-s003.png]

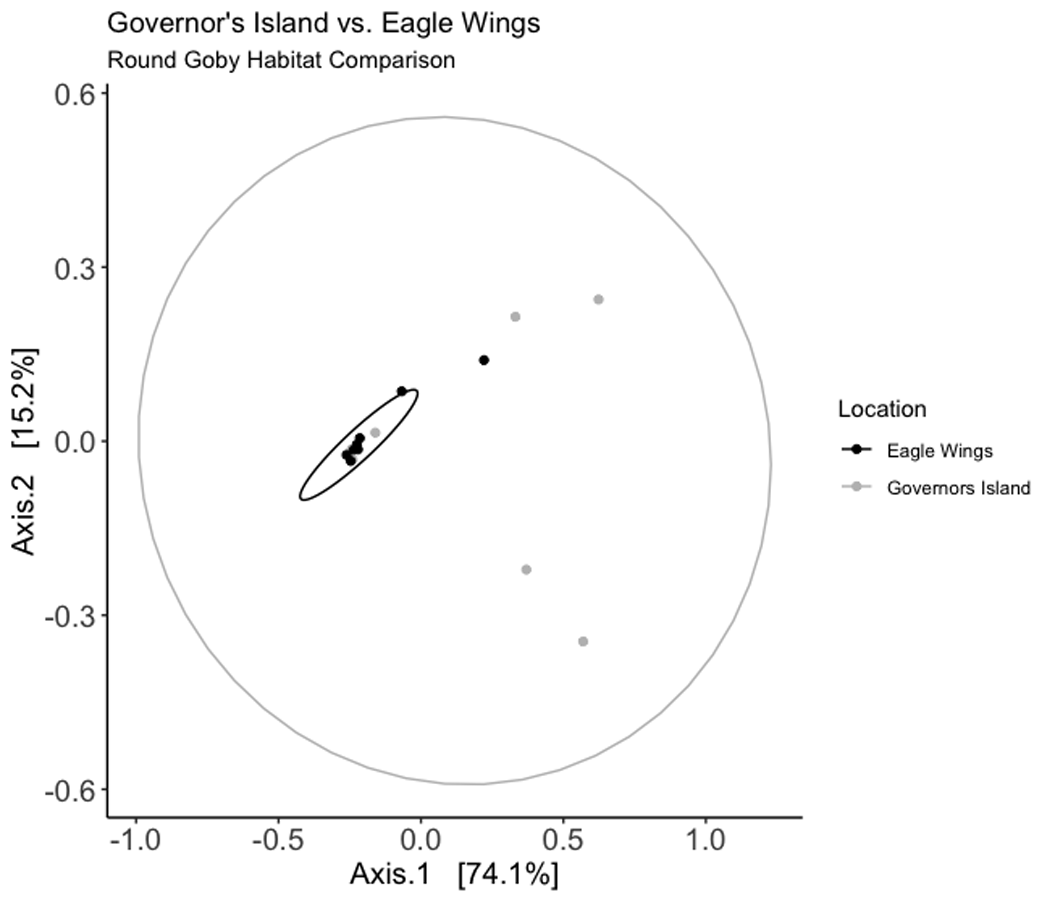

Supplement: Supplemental Information 4 — Axes 1 +2 combine for over 89% of the total data variation. The shape of the PCoA ordination closely mimics that of the habitat NMDS ordination calculated using Bray-Curtis distances. [file peerj-08-10237-s004.png]
