# Supplementary material for: Use of next generation sequencing to compare simple habitat and species level differences in the gut microbiota of an invasive and native freshwater fish species
Source: PeerJ. 2020 Dec 18;8:e10237. doi: 10.7717/peerj.10237 (PMC7751434; doi:10.7717/peerj.10237)
Supplement: Supplemental Information 5 [file peerj-08-10237-s005.docx]

| ID | Date of Capture | Location | Total Length (mm) | | Total Weight (g) |
| --- | --- | --- | --- | --- | --- |
| RG025 | 9/30/2017 | Governor’s Island | | 152 | 54.3 |
| RG026 | 9/30/2017 | Governor’s Island | | 172 | 81.0 |
| RG027 | 9/30/2017 | Governor’s Island | | 176 | 85.0 |
| RG029 | 9/30/2017 | Governor’s Island | | 160 | 76.2 |
| RG031 | 9/30/2017 | Eagle Wings | | 100 | 13.5 |
| RG032 | 9/30/2017 | Eagle Wings | | 125 | 29.5 |
| RG033 | 10/01/2017 | Eagle Wings | | 110 | 19.4 |
| RG034 | 10/01/2017 | Eagle Wings | | 138 | 37 |
| RG037 | 10/07/2017 | Eagle Wings | | 124 | 32.2 |
| RG038 | 10/07/2017 | Eagle Wings | | 112 | 23.1 |
| RG043 | 10/07/2017 | Eagle Wings | | 88 | 9 |
| RG045 | 10/07/2017 | Eagle Wings | | 105 | 16.8 |
| RG048 | 10/08/2017 | Governor’s Island | | 167 | 77.9 |
| RG049 | 10/08/2017 | Governor’s Island | | 96 | 12.5 |
| RG050 | 10/10/2017 | Governor’s Island | | 95 | 11.6 |
| YBH039 | 10/07/2017 | French Creek | | 125 | 23.2 |
| YBH041 | 10/07/2017 | French Creek | | 143 | 35.8 |
| YBH051 | 10/10/2017 | French Creek | | 173 | 56.3 |
| YBH052 | 10/10/2017 | French Creek | | 195 | 86.4 |
| YBH053 | 10/10/2017 | French Creek | | 180 | 66.1 |
| YBH055 | 10/10/2017 | French Creek | | 140 | 36.2 |
| YBH056 | 10/10/2017 | French Creek | | 170 | 59.1 |

**Supplementary Table S1:** Collection information pertaining to the 15 Round Goby (RG) and 7 Yellow Bullhead (YBH) captured along the St. Lawrence River and its tributaries during Fall 2017.
