# Supplementary material for: Use of next generation sequencing to compare simple habitat and species level differences in the gut microbiota of an invasive and native freshwater fish species
Source: PeerJ. 2020 Dec 18;8:e10237. doi: 10.7717/peerj.10237 (PMC7751434; doi:10.7717/peerj.10237)
Supplement: Supplemental Information 6 — The total OTU richness at the maximum sequencing depth is also given (sequencing depth for each sample is given in parenthesis). [file peerj-08-10237-s006.docx]

| ID | Slope at normalization (OTU/1000 sequences) | Richness at normalization | Richness at maximum | Difference |
| --- | --- | --- | --- | --- |
| RG025 | 0.125 | 50 | 53 (113,903) | 3 |
| RG026 | 0.215 | 42 | 57 (152,735) | 15 |
| RG027 | 0.311 | 62 | 80 (132,957) | 18 |
| RG029 | 0.091 | 18 | 31 (154,813) | 13 |
| RG031 | 0.789 | 73 | 137 (196,026) | 64 |
| RG032 | 0.319 | 67 | 84 (159,124) | 17 |
| RG033 | 0.17 | 31 | 44 (167,708) | 13 |
| RG034 | 0.038 | 25 | 27 (122,742) | 2 |
| RG037 | 0.092 | 44 | 47 (174,010) | 3 |
| RG038 | 0.555 | 83 | 83 (66,906) | 0 |
| RG043 | 0.536 | 143 | 167 (143,575) | 24 |
| RG045 | 0.267 | 111 | 111 (41,250) | 0 |
| RG048 | 0.142 | 58 | 64 (121,006) | 6 |
| RG049 | 0.392 | 115 | 127 (106,982) | 12 |
| RG050 | 0.191 | 65 | 66 (47,783) | 1 |
| YBH039 | 0.992 | 133 | 209 (215,238) | 76 |
| YBH041 | 0.578 | 75 | 116 (17,556) | 41 |
| YBH051 | 2.663 | 288 | 423 (121,145) | 135 |
| YBH052 | 1.6 | 247 | 314 (140,473) | 67 |
| YBH053 | 0.528 | 60 | 92 (152,580) | 32 |
| YBH055 | 1.79 | 225 | 324 (154,319) | 99 |
| YBH056 | 1.408 | 218 | 298 (115,309) | 70 |
