# Supplementary material for: Use of next generation sequencing to compare simple habitat and species level differences in the gut microbiota of an invasive and native freshwater fish species
Source: PeerJ. 2020 Dec 18;8:e10237. doi: 10.7717/peerj.10237 (PMC7751434; doi:10.7717/peerj.10237)
Supplement: Supplemental Information 7 — The total OTU richness at the maximum sequencing depth is also given (sequencing depth for each sample is given in parenthesis). [file peerj-08-10237-s007.docx]

| ID | Slope at normalization (OTU/1000 sequences) | Richness at normalization | Richness at maximum | Difference |
| --- | --- | --- | --- | --- |
| RG025 | 0.111 | 47 | 51 (113,623) | 4 |
| RG026 | 0.206 | 46 | 56 (152,819) | 10 |
| RG027 | 0.3231 | 58 | 68 (132,494) | 10 |
| RG029 | 0.097 | 29 | 34 (155,459) | 5 |
| RG031 | 0.813 | 81 | 141 (196,911) | 60 |
| RG032 | 0.279 | 67 | 80 (159,128) | 13 |
| RG033 | 0.15 | 30 | 43 (167,713) | 13 |
| RG034 | 0.056 | 30 | 30 (124,248) | 0 |
| RG037 | 0.074 | 41 | 45 (174,006) | 4 |
| RG038 | 0.19 | 81 | 86 (66,907) | 5 |
| RG043 | 0.485 | 145 | 164 (144,499) | 19 |
| RG045 | 0.195 | 105 | 105 (40,972) | 0 |
| RG048 | 0.139 | 58 | 63 (120,757) | 5 |
| RG049 | 0.31 | 99 | 116 (106,022) | 17 |
| RG050 | 0.213 | 65 | 67 (47,784) | 2 |
